# Neutralization of Typhoid Toxin by Alpaca-Derived, Single-Domain Antibodies Targeting the PltB and CdtB Subunits

**DOI:** 10.1128/iai.00515-21

**Published:** 2022-02-17

**Authors:** Hari P. Dulal, David J. Vance, Durga P. Neupane, Xiangcheng Chen, Jacqueline M. Tremblay, Charles B. Shoemaker, Nicholas J. Mantis, Jeongmin Song

**Affiliations:** a Department of Microbiology and Immunology, Cornell University, Ithaca, New York, USA; b Division of Infectious Diseases, Wadsworth Center, New York State Department of Health, Albany, New York, USA; c Department of Infectious Diseases and Global Health, Tufts University, North Grafton, Massachusetts, USA; Washington State University

**Keywords:** antibody, nanobody, *S.* Typhi, toxin neutralization, typhoid fever, typhoid toxin, VHH single-domain antibody, neutralizing antibodies

## Abstract

Typhoid toxin is secreted by the typhoid fever-causing bacterial pathogen Salmonella enterica serovar Typhi and has tropism for immune cells and brain endothelial cells. Here, we generated a camelid single-domain antibody (VHH) library from typhoid toxoid-immunized alpacas and identified 41 VHHs selected on the glycan receptor-binding PltB and nuclease CdtB. VHHs exhibiting potent *in vitro* neutralizing activities from each sequence-based family were epitope binned via competition enzyme-linked immunosorbent assays (ELISAs), leading to 6 distinct VHHs, 2 anti-PltBs (T2E7 and T2G9), and 4 anti-CdtB VHHs (T4C4, T4C12, T4E5, and T4E8), whose *in vivo* neutralizing activities and associated toxin-neutralizing mechanisms were investigated. We found that T2E7, T2G9, and T4E5 effectively neutralized typhoid toxin *in vivo*, as demonstrated by 100% survival of mice administered a lethal dose of typhoid toxin and with little to no typhoid toxin-mediated upper motor function defect. Cumulatively, these results highlight the potential of the compact antibodies to neutralize typhoid toxin by targeting the glycan-binding and/or nuclease subunits.

## INTRODUCTION

Typhoid toxin is a bacterial AB toxin produced by Salmonella enterica serovar Typhi (*S.* Typhi), which is expressed and secreted exclusively by *S*. Typhi after invasion of host cells (1, 2). Typhoid toxin consists of two enzymatic A subunits, CdtB and PltA, and a homopentamer of the glycan receptor-binding B subunit PltB in the pyramid-shaped heptameric A_2_B_5_ holotoxin (3). The homopentameric PltB subunits have 15 glycan-binding pockets (3 binding pockets per monomer) that are critical for multivalent, high-affinity binding of the toxin to specific glycans expressed on host cells (4, 5). PltB subunits of typhoid toxin have tropism to immune cells and brain endothelial cells on the brain-blood barrier (4). Typhoid toxin can intoxicate those immune cells recognized by PltB subunits following the glycan receptor-mediated retrograde endocytosis process in immune cells (3, 4). In contrast, after binding to brain endothelial cells, the toxin penetrates the endothelial barrier and gains access to cells in the brain, such as neuronal cells (4).

After the B subunit-mediated toxin delivery into target cells, CdtB’s nuclease activity is vital for inducing typhoid toxin-mediated cellular and *in vivo* toxicities (3–6). As such, typhoid toxin is also classified as a bacterial genotoxin. Inside target host cells, genotoxins can enter the nucleus of host cells and cause DNA damage, leading to cell cycle arrest in G_2_/M, while DNA damage repair responses are induced in host cells (7). Host cell death or senescence can occur if the DNA damage is not adequately repaired by such host responses (8–11). Using this information, we can objectively evaluate typhoid toxin-induced cellular toxicities through quantitative fluorescence microscopy by measuring host cell DNA damage repair responses and quantitative flow cytometry measuring host cell cycle arrest in G_2_/M (2–4, 6). Similarly, we can objectively quantify typhoid toxin-mediated *in vivo* toxicities using a mouse model expressing “human-like” glycans by analyzing the toxin binding to target cells, target cell DNA damage repair responses, and protection from a lethal dose typhoid toxin challenge (4).

VHH single-domain antibodies derived from camelids, often dubbed nanobodies, are the smallest available antibody-based antigen-binding fragments (2.5 nm in diameter and 4 nm in length), retaining the full binding capacity of intact antibodies (12, 13). Their compact size makes tissue and cell penetration more efficient than most IgGs, as demonstrated by using various disease models, including models for bacterial and viral infections (14–17).

As typhoid toxin intoxicates target host cells after toxin delivery, which includes brain endothelial cells and neuronal cells, we aimed to examine whether small nanobodies recognizing typhoid toxin subunits can offer protection against typhoid toxin-mediated intoxications. Currently, no intervention strategies targeting typhoid toxin are available. In this study, we generated a VHH phagemid library targeting typhoid toxin, characterized 41 VHH antibodies obtained from the library screen, and evaluated a selection of VHHs for their *in vivo* toxin-neutralizing efficacy and the mechanisms of neutralization involved.

## RESULTS

### Generation of VHH antibodies targeting PltB or CdtB subunits of typhoid toxin.

To generate VHHs targeting PltB or CdtB subunits of typhoid toxin, we immunized two alpacas (Cassie and Noo) with five doses of typhoid toxoid in the same A_2_B_5_ toxin configuration. The alpacas had serum reciprocal endpoint titers of >100,000 after two immunizations (Fig. S1 in the supplemental material). Peripheral B lymphocytes were prepared 5 days after the final immunization and used for the phagemid library construction (18). The library was screened via a two-stage process, a single low-stringency panning using 10-µg/mL CdtB or pentameric PltB subunits, followed by the second round of high-stringency panning with 1-µg/mL CdtB or pentameric PltB subunits. Thirty-four anti-PltB VHHs and 7 anti-CdtB VHHs, totaling 41 VHH antibodies, were selected based on enzyme-linked immunosorbent assays (ELISAs) for DNA sequence analysis to identify unique VHH families (Fig. S2 to S12). VHHs were grouped into families based on inferred amino acid sequence homologies in complementarity-determining region 3 (CDR3) (Fig. S2 to S12). To obtain purified VHHs for characterization, all 41 VHHs were subcloned in a pET32b-positive (pET32b^+^) expression vector, expressed in Escherichia coli, and affinity purified, as described (18).

### VHHs neutralize typhoid toxin *in vitro* with different neutralizing capabilities.

**FIG 1:**
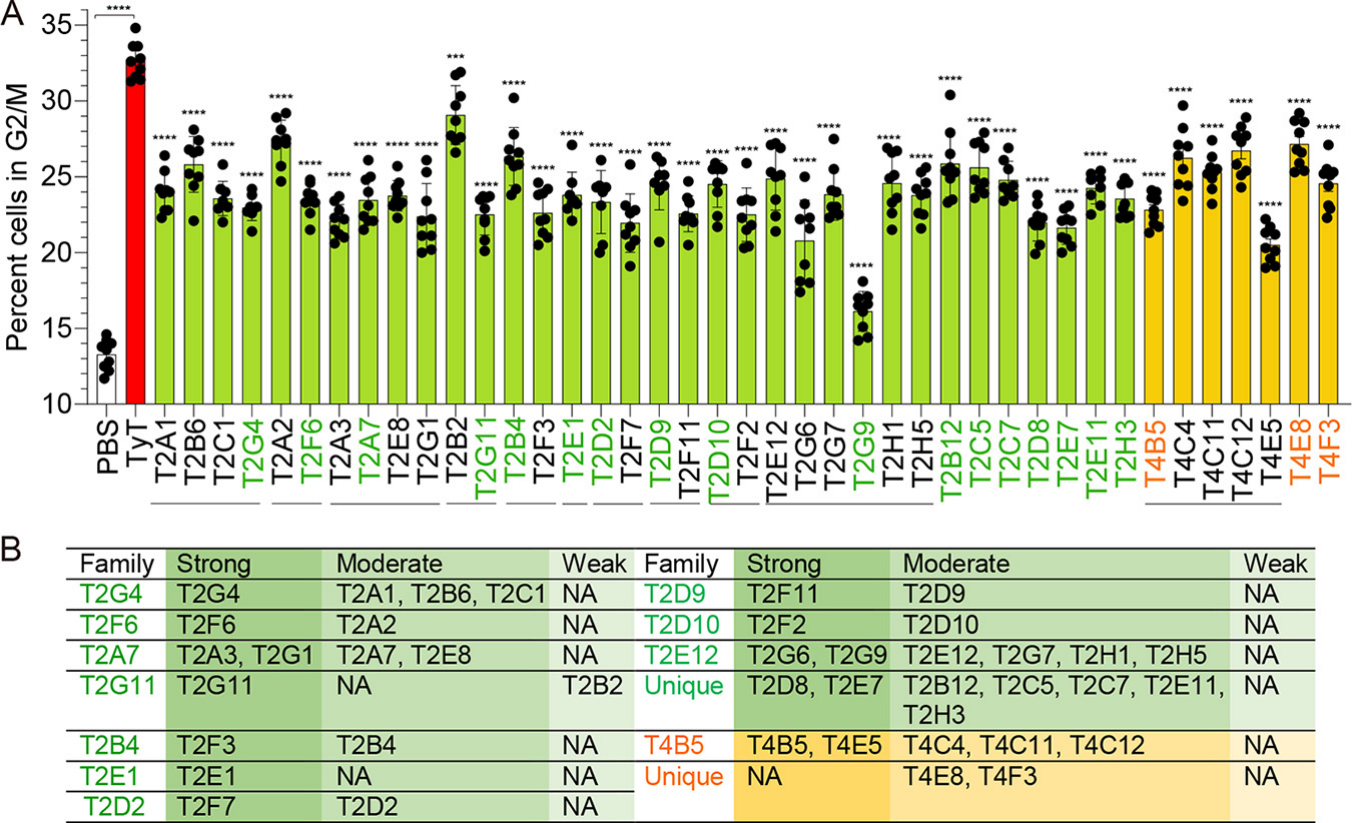
VHH antibodies generated in this study neutralize typhoid toxin *in vitro* with different neutralizing capabilities. (A) Percentage of cells in the G_2_/M cell cycle that indicates the typhoid toxin-mediated toxicity. Jurkat cells were treated with PBS, typhoid toxin (TyT; 70 pg toxin in 500 μL medium), or a mixture of TyT and each indicated VHH (70 pg toxin and 8 ng VHH in 500 μL medium) for 18 h. Cell cycle profiles were analyzed via flow cytometry. Three independent experiments were performed. Bars represent average ± SEM. ****, *P* < 0.0001. *n* = 9 per group. Unpaired two-tailed *t* tests. Solid gray lines under antibody names are to indicate VHHs in the same family. VHH representing each family is highlighted in green for anti-PltB antibodies and orange for anti-CdtB antibodies. (B) Categorizations of VHHs according to their toxin-neutralizing capabilities, strong (>50% toxin neutralization), moderate (25% to 50%), and weak (<25%). See also Fig. S2 to 12 in the supplemental material for their sequence-based family grouping and Fig. S13 for toxin neutralization in Henle-407 cells. NA, not available.

All 41 VHHs were tested for their ability to neutralize typhoid toxin *in vitro* by assessing host cell cycle profiles of Jurkat cells. Jurkat cells were treated for 18 h as previously described (4, 19) with phosphate-buffered saline (PBS), typhoid toxin (70 pg), or typhoid toxin (70 pg) premixed with each indicated VHH (8 ng per each 24-well plate). DNA contents of each treated cell were analyzed using flow cytometry. As shown in Fig. 1A, VHHs neutralized typhoid toxin, albeit with different neutralizing capabilities. T2G9 and T4E5 were the most potent among anti-PltB and anti-CdtB VHHs, respectively (Fig. 1A).

We next conducted additional *in vitro* toxin neutralization assays using Henle-407 cells for a selected set of VHHs, which exhibit an increased dynamic range for assessing toxin neutralization effects. We found that the *in vitro* toxin neutralization effect of each VHH is consistent between the two host cells utilized as screening tools (Fig. S13). Based on the *in vitro* toxin neutralization effects of VHHs, we binned VHH antibodies into three groups, strong (>50% toxin neutralization), moderate (25 to 50%), and weak (<25%) (Fig. 1B). We then selected 11 anti-PltB (T2G4, T2F6, T2G1, T2G11, T2F3, T2E1, T2F7, T2F11, T2F2, T2G9, and T2E7) from each anti-PltB antibody family group with strong toxin neutralization effects, while all 7 anti-CdtB VHHs (T4B5, T4C4, T4C11, T4C12, T4E5, T4E8, and T4F3) were included (Fig. 1B and Fig. S2 to 12) for the epitope assignment study.

### Eleven anti-PltB VHHs recognize at least four different epitopes on typhoid toxin.

**FIG 2:**
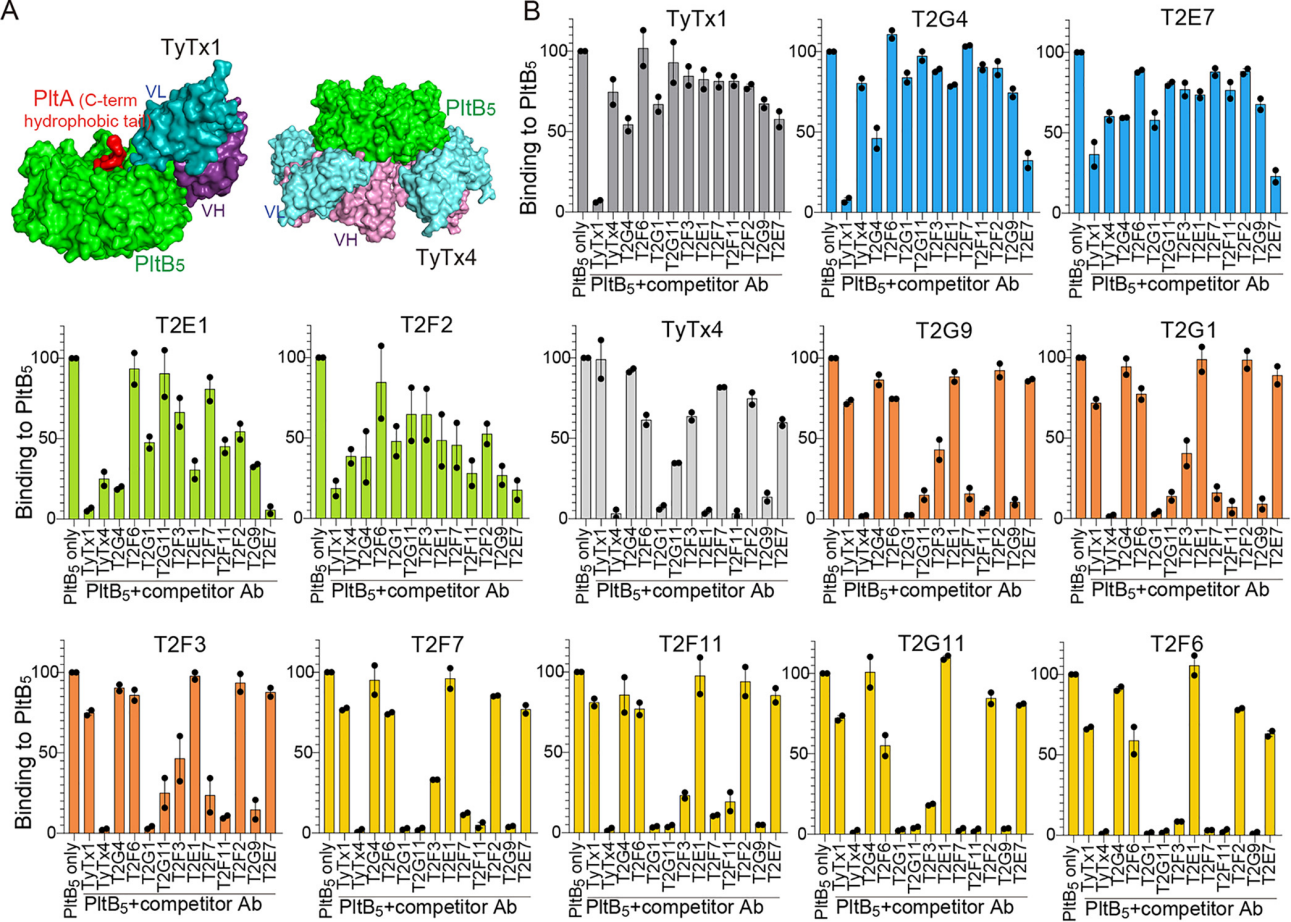
Eleven anti-PltB VHHs recognize at least four different epitopes on typhoid toxin. (A) Close-up views of the TyTx1 Fab-typhoid toxin and TyTx4-typhoid toxin complex structures solved via cryo-EM. TyTx1 variable region light chain (VL) and heavy chain (VH), dark cyan and dark purple, respectively; TyTx4 VL and VH, cyan and purple, respectively; PltB pentamer, green; PltA C-term hydrophobic tail, red. PltA and CdtB subunits are not shown. Adapted from reference 20 to explain the neutralizing epitopes recognized by TyTx1 and TyTx4 IgGs. (B) Comparative competition ELISAs of anti-PltB VHHs, TyTx1, and TyTx4. Biotinylated PltB alone (PltB_5_ only) or biotinylated PltB preincubated with indicated competitor VHH, TyTx1, or TyTx4 (PltB_5_ plus competitor Ab) were added to the ELISA plates displaying each antibody indicated on the top of the graph. VHHs exhibiting similar competition ELISA results are color-coded. Two independent experiments were performed.

Next, we determined epitope locations recognized by 11 anti-PltB VHHs through a series of competition ELISAs. We exploited two IgG monoclonal antibodies (IgGs), TyTx1 and TyTx4, as we recently determined their precise binding sites on PltB subunits through cryogenic electron microscopy (cryo-EM) (20). TyTx1 and TyTx4 recognize specific amino acid residues located on the lateral and bottom sides of pentameric PltB subunits, respectively (Fig. 2A) (20). Each individual VHH, TyTx1, and TyTx4 were immobilized on the ELISA plates, as indicated on the top of each graph in Fig. 2B. For competition ELISAs, biotinylated PltB alone (PltB_5_ only) or biotinylated PltB preincubated with indicated competitor VHH, TyTx1, or TyTx4 (PltB_5_ plus competitor Ab) were added to the ELISA plates displaying each indicated antibody (Fig. 2B). The competitive ELISA results suggest at least four distinct groups of anti-PltB VHHs, recognizing at least 4 different epitopes on PltB pentamer (color-coded in Fig. 2B). In particular, T2G4 and T2E7 show a similar competition pattern (blue), while T2E1 and T2F2 are similar (green). Furthermore, these four VHHs and TyTx1 show similar competition patterns, which are markedly different from the remaining anti-PltB VHHs, indicating that, like TyTx1, T2G4, T2E7, T2E1, and T2F2 recognize epitopes on the lateral side of the pentameric PltB subunits (Fig. 2A). Likewise, the competition profile of TyTx4 is similar to the profiles of T2G9, T2G1, and T2F3 (orange group) and T2F7, T2F11, T2G11, and T2F6 (yellow group in Fig. 2B), indicating these two groups of anti-PltB VHHs bind to epitopes located on the bottom side of the pentameric PltB subunits (Fig. 2B). Based on the epitope assignment and *in vitro* toxin neutralization efficacy, we further selected two anti-PltB VHH antibodies, T2E7 and T2G9, for *in vivo* toxin neutralization studies.

### Seven anti-CdtB VHHs recognize at least four different epitopes on typhoid toxin.

**FIG 3:**
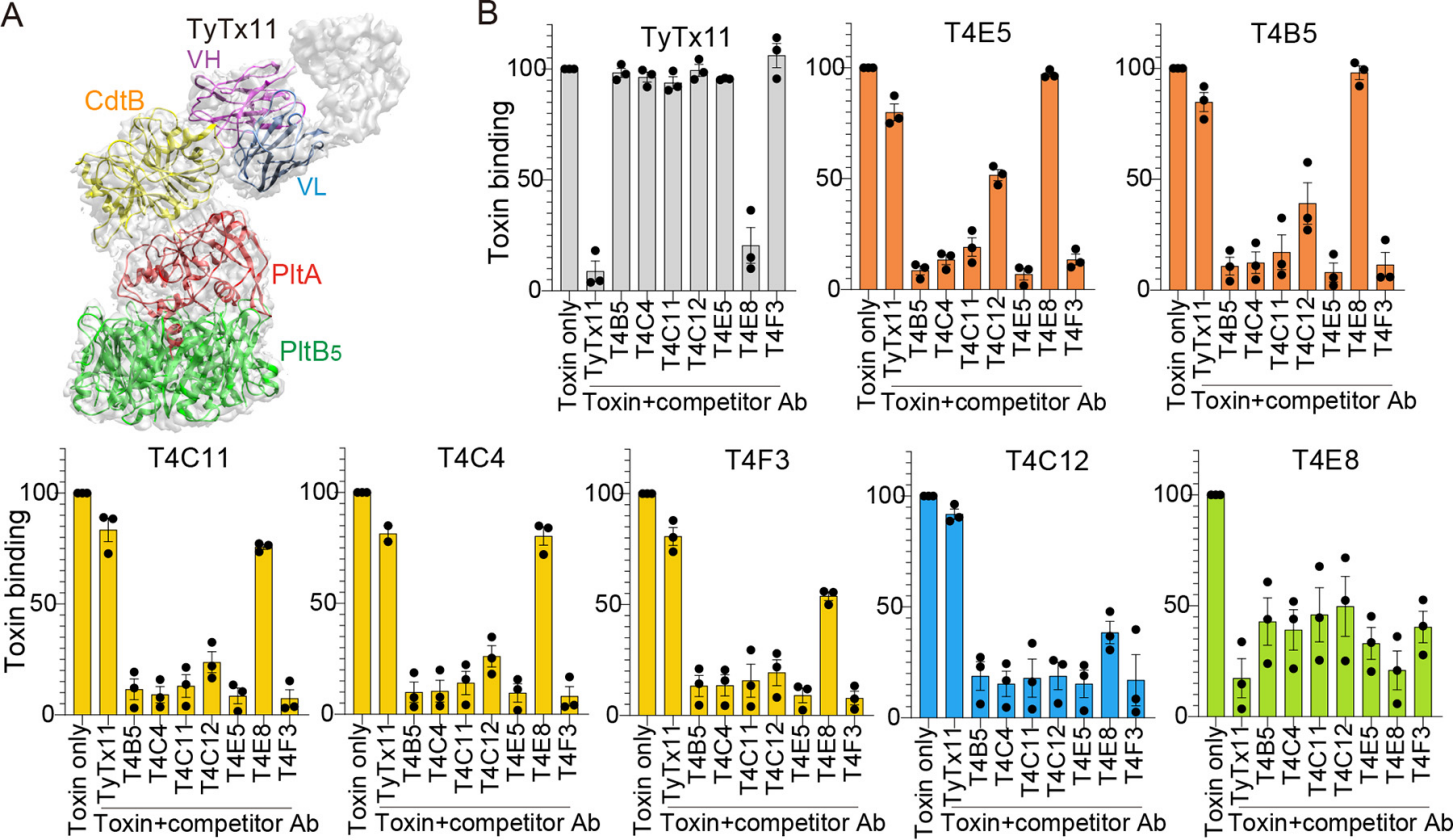
Seven anti-CdtB VHHs recognize at least four different epitopes on typhoid toxin. (A) Close-up view of the TyTx11-typhoid toxin complex structure solved via cryo-EM. TyTx11 variable region light chain (VL) and heavy chain (VH), blue and purple, respectively; PltB pentamer, green; PltA, red, CdtB, yellow. Adapted from reference 6 to explain the neutralizing epitope recognized by TyTx11 IgG. (B) Comparative competition ELISAs of anti-CdtB VHHs and TyTx11. Biotinylated typhoid toxin (toxin only) or biotinylated typhoid toxin preincubated with indicated competitor VHH or TyTx11 (toxin plus competitor Ab) were added to the ELISA plates displaying each antibody indicated on the top of the graph. VHHs exhibiting similar competition ELISA results are color-coded. Three independent experiments were performed.

We also performed competitive ELISAs for 7 anti-CdtB VHHs and TyTx11 to determine their epitope locations relative to TyTx11. TyTx11 recognizes the flexible region adjacent to the CdtB catalytic site containing the key residue His160 (Fig. 3A), which causes significant conformational changes of the flexible loop and the region adjacent to the CdtB catalytic site, resulting in the inhibition of the nuclease activity of CdtB (6). We carried out competition ELISAs for anti-CdtB antibodies using biotinylated typhoid toxin. Seven anti-CdtB VHHs showed 4 distinct competition patterns, all of which are different from the competition pattern of TyTx11, T4E5, and T4B5 in one group (orange); T4C11, T4C4, and T4F3 in another (yellow); T4C12 (blue); and T4E8 (green) (Fig. 3B). We selected T4E5, T4C4, T4C12, and T4E8 to have at least one VHH antibody from each group for *in vivo* toxin neutralization studies.

### Selected VHHs neutralize typhoid toxin *in vivo* and offer mice protection against lethal dose typhoid toxin challenge.

**FIG 4:**
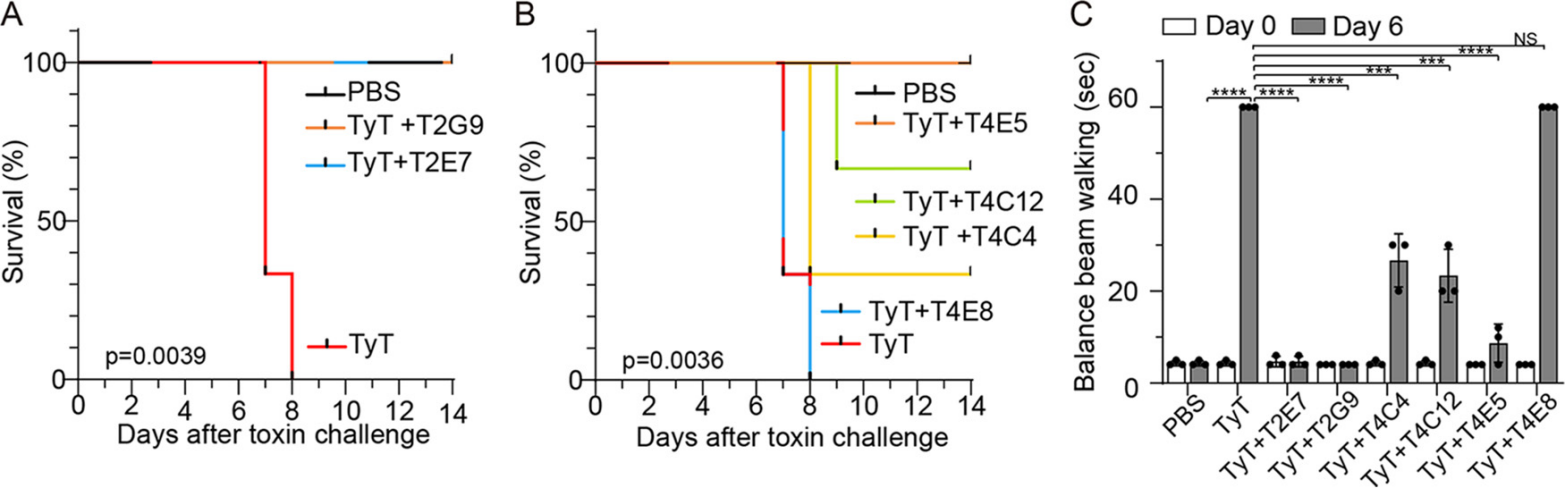
Selected VHHs neutralize typhoid toxin *in vivo* and offer mice protection against lethal dose typhoid toxin challenge. (A to C) Groups of CMAH-null mice were administered with LD_100_ of typhoid toxin (2 µg, this recombinant toxin was conjugated to His_6_) with or without VHH (8 µg). Survival (A and B) and balance beam walking results of the mice on day 6 (C) after receiving PBS, or typhoid toxin with or without indicated VHH. Bars represent the mean ± SEM. *n* = 3 to 4. Specific *P* values are indicated in panels A and B. ***, *P* < 0.001; ****, *P* < 0.0001; NS, not significant relative to the PBS group for graph C. The log-rank tests were performed for panels A and B and two-tailed unpaired *t* tests for panel C. See also Table 1 and Fig. S14 to S16 in the supplemental material.

To investigate whether T2E7, T2G9, T4E5, T4C4, T4C12, and T4E8 could neutralize typhoid toxin *in vivo* and protect mice from lethal dose typhoid toxin challenge, we intravenously administered PBS, typhoid toxin (100% lethal dose [LD_100_]), or a mixture of typhoid toxin-VHH (1:4 ratio by mass) to mice (*n* = 3). Mice were then evaluated for upper motor function defects on day 6 and monitored for survival for 14 days after toxin administration, as previously described (4). CMAH knockout (KO) mice were used, which express the human-type glycan receptor for typhoid toxin and have been established as a model for *in vivo* typhoid toxin intoxication studies (4, 19). Animals surviving beyond day 14 were considered fully protected, as previously described (4). The anti-PltB VHHs T2G9 and T2E7 afforded mice complete protection against lethal dose typhoid toxin challenge (Fig. 4A). Among anti-CdtB VHHs, T4E5 offered 100% protection to all mice, and T4C12 permitted approximately two-thirds of mice to survive from the toxin challenge, while T4C4 protected one-third of mice. T4E8 provided no protection from lethal typhoid toxin challenge (Fig. 4B).

We previously demonstrated that PltB-mediated tropism of typhoid toxin to brain endothelial cells in CMAH-null mice results in upper motor function defects, which can be objectively quantified by carrying out a balance beam walking test on day 6 (4). Like we reported previously, CMAH-null mice administered PBS took approximately 3 s to cross the balance beam. In contrast, mice that received typhoid toxin took 60 s or longer to cross the beam or failed to cross, all of which are scored as 60 s (max) in the graph (Fig. 4C). Consistent with the survival results, mice that received a lethal dose of typhoid toxin mixed with T2E7, T2G9, or T4E5 showed little to no upper motor function defects. T4C4 and T4C12 offered only partial protection from typhoid toxin-induced upper motor function defects, and T4E8 offered nearly no protection (Fig. 4C). These results indicate that T2E7, T2G9, and T4E5 are the most efficacious antitoxins, as demonstrated by 100% mouse survival and little to no upper motor function defect after a lethal dose typhoid toxin challenge (Fig. 4). In addition, we verified that the *in vivo* toxin neutralization effects of VHHs are indeed due to the VHHs by conducting additional mouse survival assays using the cleaved, size exclusion chromatography-purified T2G9 and T4E5 that contain the VHH part only without any tags (Fig. S14). These results are consistent with *in vitro* toxin neutralization data where T2G9 and T4E5 were the most efficacious toxin-neutralizing VHHs among anti-PltB VHHs and anti-CdtB VHHs, respectively (Fig. 1A).

**FIG 5:**
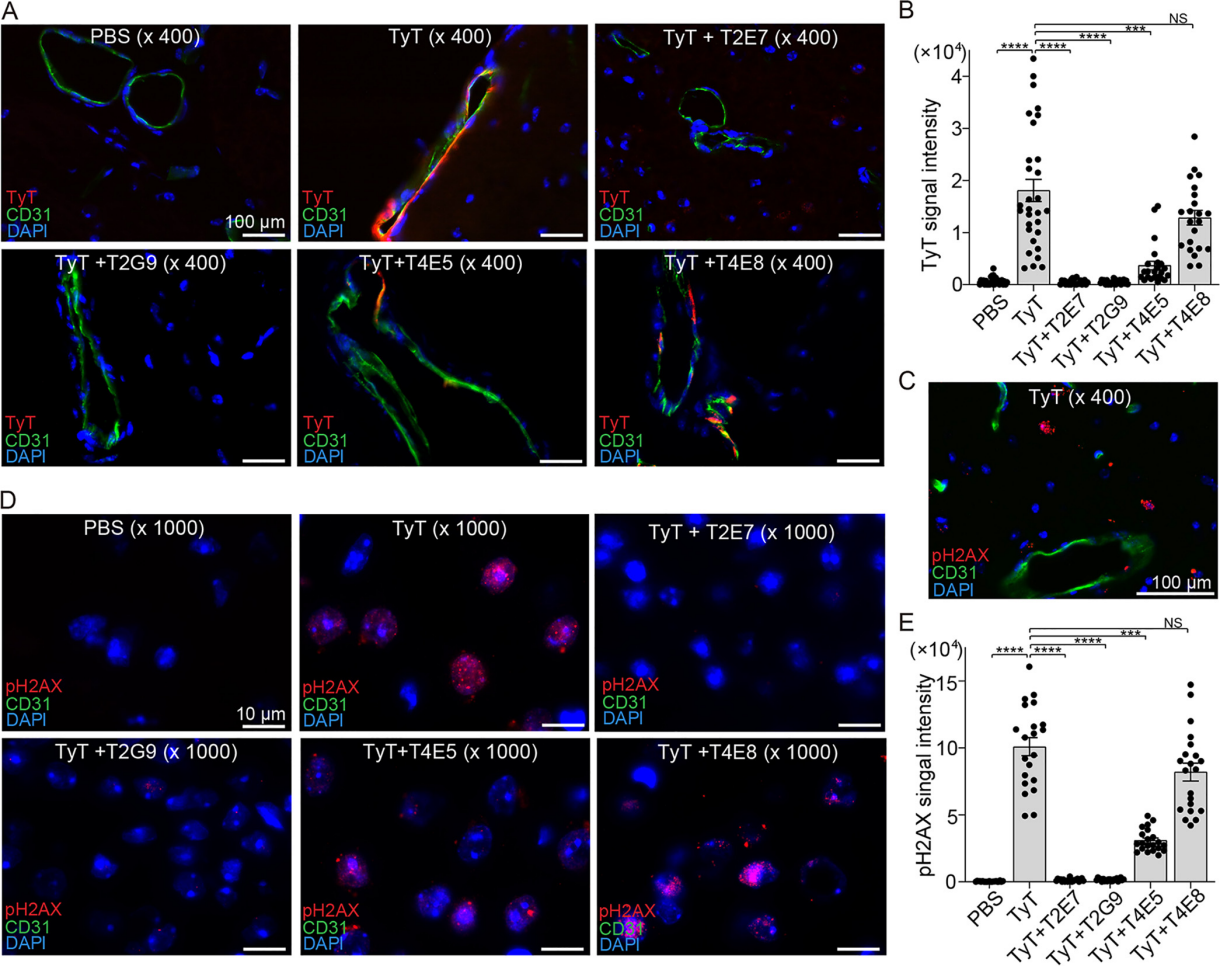
Mechanisms of typhoid toxin neutralization by VHHs. (A) Representative fluorescent images showing typhoid toxin binding or VHH-mediated inhibition of toxin binding. CMAH-null mice received PBS, AF555-typhoid toxin (TyT), or AF555-TyT plus VHH for 2 h. TyT (red), CD31 (endothelial cells), and host cell DNA (blue). Scale bar, 100 μm. (B) Typhoid toxin signal quantification of microscopic images obtained from two independent experiments. (C to D) Representative fluorescence microscope images in low (C) and high magnification (D) showing pH2AX (red, reflecting host cell DNA damage repair response), CD31 (endothelial cells), and host cell DNA (blue). Scale bar, 100 μm. (E) pH2AX signal quantification of microscopic images obtained from two independent experiments. Relative fluorescence signal intensities in panels B and E were quantified using ImageJ, as indicated in Materials and Methods. Bars in panels B and E represent average ± SEM. ***, *P* < 0.001; ****, *P* < 0.0001, relative to the PBS group or the toxin group as indicated in the graph. Two-tailed unpaired *t* tests. *n* = 2 to 3.

We determined the binding affinities of six antibodies used for *in vivo* survival assays in Fig. 4 using an Octet biolayer interferometer to see whether antibody-binding affinities contribute to the *in vivo* protection efficacies. All six antibodies exhibited at least nanomolar binding affinities to typhoid toxin (Table 1 and Fig. S15). T4E5, the most potent anti-CdtB VHH, showed a picomolar binding affinity to toxin, which is much higher than the binding affinities of the remaining anti-CdtB VHHs, T4C4, T4C12, and T4E8 (Table 1 and Fig. S15), indicating that the binding affinity might play a significant role in the increased *in vivo* toxin neutralization efficacy. However, the affinities and toxin neutralization efficacies of T4C12 and T4C4 did not exhibit a straightforward correlation between the two (Table 1 and Fig. 4B), indicating that additional factors also contribute to the observed *in vivo* protection efficacies. Moreover, it is worthwhile to note that we obtained data indirectly indicating that anti-CdtB VHHs (e.g., T4E5, T4E8) complexed with typhoid toxin Alexa Fluor 555 likely remained in animals at 24 h after administration, as supported by a significant portion of peripheral lymphocytes that were positive to typhoid toxin-VHH (Fig. S16) combined with data shown in Fig. 4 and 5. These data indirectly support the concept that the large size of the toxin-VHH complex contributes to the increased *in vivo* remaining pharmacokinetics of VHHs.

**TABLE 1:** Antityphoid toxin VHH-binding affinities^*c*^

VHH^*a*^	Target	*K*_on_ (M^−1^s^−1^)^*b*^	*K*_off_ (s^−1^)^*b*^	*K_D_* (M)^*b*^
**T2E7**	PltB	4.43 × 10^5^, 5.07 × 10^5^, 4.05 × 10^5^	2.78 × 10^−4^, 3.13 × 10^−4^, 3.69 × 10^−4^	6.29 × 10^−10^, 6.18 × 10^−10^, 9.11 × 10^−10^
**T2G9**	PltB	1.55 × 10^5^, 1.39 × 10^5^, 1.05 × 10^5^	3.87 × 10^−5^, 4.75 × 10^−5^, 1.01 × 10^−6^	2.50 × 10^−10^, 3.41 × 10^−10^, 9.59 × 10^−12^
T4C4	CdtB	9.29 × 10^5^, 2.36 × 10^6^	2.64 × 10^−4^, 2.60 × 10^−4^	2.84 × 10^−10^, 1.10 × 10^−10^
T4C12	CdtB	4.26 × 10^5^, 1.56 × 10^6^	1.21 × 10^−3^, 2.43 × 10^−3^	2.84 × 10^−9^, 1.55 × 10^−9^
**T4E5**	CdtB	4.24 × 10^5^, 3.82 × 10^5^, 3.06 × 10^5^	<1 × 10^−7^, 2.50 × 10^−5^, 2.93 × 10^−5^	<1 × 10^−12^, 6.54 × 10^−11^, 9.58 × 10^−11^
T4E8	CdtB	5.33 × 10^4^, 7.36 × 10^4^	1.08 × 10^−4^, 3.27 × 10^−4^	2.03 × 10^−9^, 4.44 × 10^−9^

a VHHs in bold exhibit the strongest neutralization effects *in vivo*.

b Individual values of 2 to 3 experiments. A 1:1 model was used to calculate the affinities. *K_D_*, equilibrium dissociation constant; *K*_on_, association rate constant; *K*_off_, dissociation rate constant.

c See also Fig. S15 in the supplemental material for Octet affinity sensorgrams.

### Mechanisms of typhoid toxin neutralization by VHHs.

To understand the neutralizing mechanisms of T2E7, T2G9, and T4E5, we assessed VHH-mediated inhibition of typhoid toxin binding to target cells 2 h after toxin administration and toxin-induced host cell DNA damage repair responses on day 6 after toxin administration. For the typhoid toxin-binding inhibition assay, animals were administered Alexa Fluor 555 (AF555)-conjugated typhoid toxin (red) with or without VHH and perfusion sacrificed 2 h after toxin administration, and we processed the brain tissues for immunofluorescence, as previously described (4) (Fig. 5A and B). DAPI (4′,6-diamidino-2-phenylindole) and CD31 were included to stain all brain cells (blue nuclei) and endothelial cells (green), respectively (Fig. 5A). T2E7 and T2G9 completely inhibited typhoid toxin binding to brain endothelial cells, whereas T4E5 and T4E8 resulted in partial inhibition and little to no inhibition of the typhoid toxin binding to brain endothelial cells, respectively (Fig. 5B). Next, we administered a separate group of CMAH-null mice with a lethal dose of typhoid toxin with or without VHH, perfusion sacrificed them 6 days after toxin administration, and processed the brain tissues to evaluate host DNA damage repair responses (Fig. 5C to E). Our previous study indicated that brain endothelial cells recognized by PltB serve as an entry point for the toxin to cells in the brain (4). Consistent with our previous finding (4), as shown in the lower magnification image (Fig. 5C), we found that neuronal cells, but not endothelial cells (CD31^+^) and other cells encircling CD31^+^ cells such as smooth muscle cells, astrocytes, and pericytes, exhibited a robust pH2AX signal (red), signifying cells undergoing host cell DNA damage repair response in a typhoid toxin treatment-dependent manner (Fig. 5C). Consistent with other data, the addition of T2E7 and T2G9 to the lethal dose typhoid toxin permitted host cells to remain at background levels of the host DNA damage repair response, similar to the level observed in PBS-treated mice (Fig. 5D and E). T4E5 markedly reduced the host responses, indicating the protective role of T4E5 from typhoid toxin-induced intoxication on these cells. In contrast, unlike T2E7, T2G9, and T4E5, T4E8 failed to protect mice from typhoid toxin-induced intoxication on these cells (Fig. 5D and E).

## DISCUSSION

We generated and characterized 41 typhoid toxin-neutralizing VHH antibodies, consisting of 34 antibodies targeting the glycan-receptor binding subunit PltB and 7 antibodies targeting the nuclease subunit CdtB. Both PltB and CdtB of typhoid toxin have been demonstrated to be essential for typhoid toxin-mediated *in vitro* and *in vivo* toxicities, as the switching of a single amino acid residue critical for either glycan-receptor binding on PltB or nuclease activity on CdtB, to alanine abolished the toxicity of typhoid toxin (3–5, 19, 21). We purified these 41 VHH antibodies targeting either PltB or CdtB and characterized them using a series of *in vitro* and *in vivo* assays in this study, enabling us to conclude that 3 nanobodies, T2E7, T2G9, and T4E5, are the most efficacious in neutralizing typhoid toxin *in vitro* and *in vivo*.

Nanobodies have several benefits over most IgGs. The compact size of VHHs is advantageous for antibody delivery to the target sites compared to most IgGs. Moreover, well-established genetic engineering strategies for generating multivalent VHHs targeting multiple epitopes on antigens make the engineering of multivalent VHHs straightforward (22–24), which is anticipated to be useful for future studies using T2E7, T2G9, and T4E5. For instance, we recently found that it is not straightforward to directly mask all 15 glycan receptor-binding pockets available per holotoxin using IgGs because of the A subunit-mediated interference with IgG binding to the laterally located epitopes on PltB subunits (20). It is anticipated that a mixture of several VHHs recognizing glycan receptor-binding pockets located on the lateral and bottom sides or a single engineered multivalent VHH can mask all 15 glycan receptor-binding pockets. Future studies on this aspect involving a series of cryo-EM-assisted structure and function analyses would address whether this prediction is correct.

We carried out comparative epitope mapping studies for 41 VHHs by exploiting IgGs whose specific epitopes were recently determined by cryo-EM, TyTx1, TyTx4, and TyTx11 (6, 20). The competition pattern of T2E7 over each VHH, TyTx1, and TyTx4 was similar to that of TyTx1 (Fig. 2), suggesting that, like TyTx1, T2E7 recognizes amino acids located on/near the glycan receptor-binding pocket 1 essential for its binding to α2-3 and α2-6 sialosides (5, 25). Likewise, the competition patterns of T2G9 and TyTx4 were alike (Fig. 2), suggesting that, like TyTx4, T2G9 recognizes the region on/near the glycan-receptor binding pockets 2 and 3 that are located on the bottom side of PltB, critical for its binding to α2-3 sialosides (5, 25). Despite the similarities, we predict that the specific epitopes recognized by T2E7 and T2G9 are not identical to TyTx1 and TyTx4, respectively, as their competition ELISA results are not identical. All 7 anti-CdtB VHH antibodies, including the most efficacious T4E5, do not seem to recognize the exact amino acids recognized by TyTx11, as their competition ELISA patterns are different (Fig. 3). Through cryo-EM, future epitope mapping studies would determine the precise amino acid residues recognized by T2E7, T2G9, and T4E5, expanding the panel of typhoid toxin-neutralizing epitopes from the ones identified from the IgG studies and increasing the chance of masking all critical toxin residues.

Unlike many other virulence factors and genes conferring antibiotic resistance, typhoid toxin genes are highly conserved across all clinical isolates of *S.* Typhi, as they harbor 100% identical typhoid toxin genes (5, 6, 20). This observation supports the idea that neutralizing VHH antibodies identified from this study can neutralize typhoid toxin produced by *S.* Typhi clinical isolates. Like other bacterial pathogens with public health relevance, multidrug-resistant (MDR) and extensively drug-resistant (XDR) *S.* Typhi is widespread globally (26–30). We recently formally demonstrated that typhoid toxin is continuously secreted by antibiotic-resistant *S.* Typhi even when *S.* Typhi-infected host cells are treated with antibiotics (6). In this regard, it is anticipated that the information obtained from this study could help mitigate some aspects of typhoid toxin-mediated toxicities in the future.

Our *in vivo* protection results after a single-dose VHH administration are considered quite remarkable (Fig. 4) given the known rapid nanobody clearance kinetics. To obtain insights into the reason why these antityphoid toxin VHHs behave differently from the most conventional VHHs, we attempted to measure *in vivo* antitoxin nanobody and toxin clearance rates using the CMAH-null mouse model, but we were unable to get a straightforward answer to this question. The goal of the conducted animal experiments was to detect and quantify VHH and VHH-toxin complex present in urine, plasma, and peripheral blood lymphocytes 30 min, 2 h, and 24 h after *in vivo* administration to evaluate if VHH is rapidly removed due to its small size, whereas VHH bound to typhoid toxin remains in animals for an extended period. In addition, a new ELISA was established to specifically detect VHH, which generated a strong signal originating from known concentrations of positive controls, but not from the urine and plasma samples (data not shown), indicating that the ELISA system established was functional but VHH concentrations presented in the urine and plasma samples were below the detection limit. Although we speculated the VHH-toxin complex was able to avoid the kidney- and urination-mediated clearance and therefore remain in animals for an extended period due to its larger size, our attempt was not successful. We predict that developing an ultrasensitive detection method would help overcome the technical challenges that we had in our experimental setup. Nonetheless, it is worthwhile to note that we obtained data indirectly indicating that anti-CdtB VHHs (e.g., T4E5, T4E8) complexed with typhoid toxin-Alexa Fluor 555 likely remained in animals at 24 h after administration, as supported by a significant portion of peripheral lymphocytes was positive to typhoid toxin-VHH (Fig. S16) and data shown in Fig. 4 and 5, indirectly supporting the concept that the large size of the toxin-VHH complex contributes to the increased *in vivo* remaining pharmacokinetics of VHHs. Furthermore, through determining the binding affinities of six VHHs used for the *in vivo* protection studies, we found that the binding affinity, as well as additional unknown factor(s), likely contribute to the observed remarkable *in vivo* protection efficacies.

In summary, typhoid toxin intoxicates target host cells after toxin delivery, which includes brain endothelial cells and neuronal cells, but no intervention strategies targeting typhoid toxin are currently available. This study demonstrates that T2E7, T2G9, and T4E5 nanobodies are powerful in neutralizing typhoid toxin in *in vitro* cell and *in vivo* mouse models. The findings from this study hold promise for interventions against typhoid toxin in tissues challenging to reach, such as the brain.

## MATERIALS AND METHODS

### Ethics statement.

All animal experiments were conducted in accordance with the guidelines approved by the University’s Institutional Animal Care and Use Committee (Cornell IACUC protocol no. 2014-0084 for mouse experiments and Tufts IACUC protocol no. G2017-18 for alpaca immunizations).

### Typhoid toxin and inactive toxoid preparations.

Typhoid toxin, CdtB, PltB homopentamer, and inactive typhoid toxoid were prepared as previously described (6, 20). Note that typhoid toxin has a hexahistine tag on the C-terminal end of CdtB, which is known to reduce the CdtB-mediated toxicity by approximately 20-fold.

### Generation of the VHH phagemid library targeting typhoid toxin.

### (i) Library generation.

Two alpacas (Vicugna pacos, named Cassie and Noo) received five subcutaneous immunizations at approximately 3-week intervals with a priming immunization of 174 µg of typhoid toxoid and four booster immunizations of 100 µg. Five days following the final boost, peripheral lymphocytes from the immunized alpacas were prepared to generate the VHH phagemid library (referred to as CaNoo) as previously described (18). The estimated library complexity was 1.2 × 10^7^ independent clones of which >95% contained VHH inserts. The VHH-pIII phagemid library was maintained in E. coli TG1 and stored at −80°C.

### (ii) Library screening.

The library was subjected to two rounds of panning. Before the first round, an aliquot of the CaNoo phagemid library was grown in 100 mL superbroth (SB) supplemented with 100 μg/mL carbenicillin and 2% glucose for 2 h until the optical density at 600 nm (OD_600_) reached ∼0.6 before adding 1 mL of the VCSM13 helper phage to the culture. After 2 h, the infected bacteria were spun down (10 min, 8,000 rpm, ∼10,950 × *g*), and the pellet was reconstituted in 100 mL SB supplemented with 100 μg/mL carbenicillin, 70 μg/mL kanamycin, and 0.1% glucose overnight at 37°C. The following day, after bacteria were spun down (10 min, 8,000 rpm, ∼10,950 × *g*), and the phages in the supernatant were precipitated for 2 h at 4°C with the addition of 1:5 volume of a solution containing 20% polyethylene glycol 8000 (PEG 8000) and 2.5 M NaCl. This was then spun down (20 min, 8,000 rpm, ∼10,950 × *g*) and reconstituted in 1 mL PBS for use in panning. The phages were then panned for VHHs that were specific for either PltB or CdtB. In the first round, PltB and CdtB were immobilized on separate Immuno tubes (Nunc) overnight at a concentration of 10 μg/ml and blocked for 2 h with 2% goat serum in PBS containing 0.1% Tween 20 (PBS-T). The phagemid library was diluted 2-fold in PBS-T containing 2% goat serum, and 500 μL was exposed to each immobilized antigen for 1 h. Unbound phages were washed away with 0.1% PBS-T, and bound phages were eluted first with E. coli ER2738 (New England Biolabs) for 15 min, followed by 10 min of 0.2 M glycine, pH 2.2, which was then neutralized with 75 μL of 1 M Tris-HCl, pH 9.1. The two elutions for each screen were combined and plated onto SB plates. After overnight growth, the lawn formed was scraped and stored in SB containing 100 µg/mL carbenicillin, 2% glucose, and 15% glycerol at −80°C. An aliquot was then used to prepare phages for the second round of panning, which was the same as explained above except for the following. Immuno tubes were coated with 1 μg/mL PltB or CdtB, the phages were diluted 200-fold in PBS-T before exposure to the immobilized antigens, the exposure time of phages to antigens was 15 min, 0.5% PBS-T was used to wash away the unbound phages, and an aliquot of each elution was serially diluted 10-fold and plated.

### (iii) Hit validation.

VHH colonies were validated for their specificity to CdtB or PltB by ELISAs. In brief, individual colonies from the two-stage screening described above were then cultured overnight in 200 μL SB containing 100 μg/mL carbenicillin, 10 μg/mL tetracycline, and 2% glucose. Ten-microliter cultures were transferred to a new 96-well plate containing 160 μL SB containing 100 μg/mL carbenicillin and 10 μg/mL tetracycline, grown for 4 h until the OD_600_ reached ∼0.6, added with 70 μL of 10 mM IPTG (isopropyl-β-d-thiogalactopyranoside; final concentration, ∼3 mM IPTG), and cultured overnight to overexpress the VHH-pIII fusion proteins carrying the E-tag. The cultures were spun down the next day, and the supernatants were subjected to ELISAs for PltB and CdtB. Bound VHHs were detected with an anti-E-tag-horseradish peroxidase (HRP) secondary antibody (Bethyl Labs), developed with SureBlue TMB (KPL), and OD_450_ values were read using a VersaMax microplate reader (Molecular Devices). Positive binding clones were cultured, stored, miniprepped, and sequenced.

### VHH sequence analysis.

The positive binding clones were sequenced via Sanger sequencing. DNA sequences were translated using the LaserGene software (DNAStar). CDR3s of VHH amino acid sequences were compared to assign VHHs into families also using LaserGene. Based on the LaserGene analysis data, Fig. S2 and 12 were prepared using a series of ClustalW analyses of VHHs in each family.

### Subcloning, overexpression, and purification of VHHs.

### (i) Subcloning.

After sequencing, chosen VHHs were digested out of the phagemid vector (JSC; GenBank accession no. EU109715.1) using the restriction enzymes AscI and NotI, analyzed on a 1% TBE DNA agarose gel, excised, extracted, and ligated into a pET32b^+^ expression vector (JEG-3). The ligated plasmids were transformed into E. coli Top10 competent cells (Thermo Fisher Scientific), plated, and validated for the presence of the VHH inserts via Sanger sequencing.

### (ii) Expression.

The sequence-confirmed plasmids were transformed into E. coli Rosetta-gami 2(DE3)pLacI competent cells (Millipore Sigma) and grown overnight on LB agar plates containing 100 μg/mL carbenicillin, 34 μg/mL chloramphenicol (for pLacI, also harboring the rare E. coli tRNAs), and 2% glucose (LB-CB-CM-Glu). A single colony was picked, cultured in LB-CB-CM-Glu broth overnight, used to seed a subculture of 130 mL of LB-CB-CM, added with 1 mL of 135 mM IPTG (final concentration, 1 mM IPTG) when the culture reached the log phase, and incubated overnight at 15°C.

### (iii) Purification.

The bacteria were lysed using BugBuster Plus Lysonase kit (EMD Millipore; catalog no. 71370). His_6_-tagged thioredoxin-VHH fusion protein in the supernatant was purified using Ni-nitrilotriacetic acid (Ni-NTA) agarose (Thermo Fisher Scientific) and eluted with 250 mM imidazole. Purified VHH was dialyzed overnight in a 7-kDa-cutoff Slide-A-Lyzer dialysis cassette (Thermo Fisher Scientific) against 4 L PBS with at least one buffer change. Dialyzed protein was sterilized using a Whatman Puradisc 0.2-μm filter (Thermo Fisher Scientific) and analyzed for purity and concentration.

### (iv) Preparation of cleaved VHH.

The removal of the thioredoxin tag from thioredoxin-VHH fusion protein was performed by using Thrombin cleavage kit following the instructions from the manufacturer (Sigma-Aldrich; catalog no. RECOMT). Briefly, up to 1 mg of the total fusion protein (thioredoxin-T2G9/T2E7/T4E5) was digested for 2 h at room temperature in 1 mL thrombin-agarose resin resuspended in a buffer containing 50 mM Tris-HCl (pH 8.0) with 10 mM CaCl_2_. The cleaved VHH was recovered by centrifugation at 500 × *g* for 5 min, and the cleavage was assessed by SDS-PAGE, further subjected to size exclusion chromatography.

### VHH affinity measurements.

VHH affinity was measured on an Octet Red96e biolayer interferometer (Sartorius) using the Data Acquisition 12.0 software. Streptavidin-coated sensors (catalog no. SA; Sartorius) were used to capture biotinylated typhoid toxin at a concentration of 2 µg/mL in 2% PBS-bovine serum albumin (BSA) (buffer) for 5 min. After 3 min of baseline in buffer, sensors were then exposed to a dilution series of VHH starting at 200 nM and diluting 2-fold six times to 3.125 nM. An eighth typhoid toxin-coated sensor was exposed to buffer only and was used as a reference sample to subtract from each VHH sample to account for background drift. Association proceeded for 5 min, after which the sensors were dipped in buffer alone to measure dissociation for 30 min. The experiment was performed at 25°C, and the sample plate was continuously shaken at 1,000 rpm. For each VHH, new sensors were coated with biotinylated typhoid toxin rather than regenerating the sensors, as the low-pH glycine buffer designed to disrupt VHH-toxin interaction would also disrupt the noncovalent interaction between the PltA-CdtB heterodimer and the PltB pentamer. The raw data were then analyzed with the Data Analysis HT 12.0 software. The data were aligned to the last few seconds of the baseline signal, and the binding of each VHH was modeled as a 1:1 interaction with the toxin. Each VHH’s affinity was measured at least twice, with the three neutralizing VHHs being tested three times. All kinetic values obtained for each VHH are shown in Table 1.

### *In vitro* toxin neutralization assay.

Quantitative flow cytometry measuring host cell cycle arrest in G_2_/M was carried out to evaluate antibody-mediated neutralization of typhoid toxin-induced cellular toxicities. Jurkat cells were maintained in RPMI 1640 containing 10% fetal bovine serum (FBS) (VWR Seradigm Premium FBS; catalog no. 97068-085, which contains a slightly increased level of Neu5Gc compared to HyClone FBS [catalog no. SH30396.03; lot no. AD14962284] that we used for other reported studies), 1 mM sodium pyruvate, and 10 mM HEPES, and 1.5 × 10^5^ cells per well were seeded into 24-well plates using 500 μL medium. Typhoid toxin (70 pg) with or without VHH (8 ng in 500 μL medium) and PBS buffer only were prepared 30 min before the treatment. Jurkat cells were treated with prepared toxin and antibody mixtures, incubated for 18 h in a cell culture incubator, harvested, washed, and fixed for 2 to 3 h at −20°C in PBS containing 70% ethanol. Fixed and permeabilized cells were washed with PBS; resuspended in 500 μL PBS containing 50 μg/mL propidium iodide, 0.1 mg/mL RNase A, and 0.05% Triton X-100; incubated at 37°C for 40 min; washed with PBS; resuspended in 500 μL PBS; filtered; and read using BD Accuri C6 Plus (BD Biosciences). The results were analyzed using FlowJo software (Treestar Inc.). When indicated, Henle-407 cells were treated with PBS, typhoid toxin (TyT; 1.2 pM), or a mixture of TyT and each VHH indicated (1:400) for 66 h. Cell cycle profiles were analyzed via flow cytometry.

### Competition ELISA.

### (i) Preparation of biotinylated samples.

Biotinylated PltB and biotinylated typhoid toxin were prepared using EZ-link NHS-Biotin (Thermo Fisher Scientific; catalog no. 20217) following the vender’s recommendation. Biotinylation of PltB and typhoid toxin was completed on ice for 2 h, and the unbound biotin in the samples was removed using Amicon columns with a 10-kDa cutoff (Thermo Fisher).

### (ii) Competition ELISA.

We coated 96-well ELISA microplates (Greiner Bio-One; catalog no. 655001) were coated with indicated VHHs or monoclonal antibodies (MAbs) by incubating overnight at 4°C with 100 μL of 50 mM carbonate-bicarbonate buffer, pH 9.6, containing 1 μg/mL VHH or indicated monoclonal antibodies (TyTx1, TyTx4, and TyTx11). Wells were washed with PBS containing 0.05% Tween 20, blocked with PBS containing 1% BSA for 1 h at 37°C, washed, added with 10 ng/mL biotinylated PltB (for anti-PltB VHHs) or biotinylated typhoid toxin (for anti-CdtB VHHs) ± 1 μg/mL indicated competitor VHH, and incubated for 2 h at 37°C. Biotinylated PltB-VHH or biotinylated typhoid toxin was mixed with indicated VHH in 100 μL PBS/0.5% BSA and incubated for 30 min at 37°C before adding the mixture to the well of ELISA plates. After washing, the wells were added with 100 μL of 1:5,000 diluted Precision Protein StrepTactin-HRP conjugate (Bio-Rad; catalog no. 1610381) in PBS-0.5% BSA, incubated for 1 h at 37°C, washed, added with tetramethylbenzidine (Sigma), incubated for 10 to 30 min, and, lastly, added with 100 μL of 1 M H_3_PO_4_ to stop the color development. The results were read using a Tecan Infinite 200 Pro microplate reader.

### *In vivo* toxin neutralization assay.

A mouse survival assay was conducted to evaluate the toxin-neutralizing activities of selected VHHs. Age- and sex-matched 5- to 8- week-old CMAH-null mice were randomly allocated to each group (*n* = 3). The mice used in this study were initially purchased from the Jackson Laboratory and bred in a vivarium in the animal facility at Cornell University. All the knockout mice used were genotyped regularly. Groups of mice were injected retro-orbitally with 100-μL solutions containing 2 μg typhoid toxin (His_6_ on the CdtB subunit) only or a mixture containing typhoid toxin and each VHH (8 μg, preincubated for 30 min). Changes in the weight and survival of the toxin-injected mice were closely monitored for 14 days as previously described (3, 4, 19). Toxin-mediated motor function deficits were evaluated by a balance beam test as previously described (4).

### Immunofluorescent staining.

### (i) Mouse experiment.

For fluorescence microscopy, two mice (*n* = 2 to 3) were randomly selected for studying the VHH antibodies' roles in inhibiting the binding of typhoid toxin to brain endothelial cells and host cell DNA damage repair responses. Brain samples were harvested 2 h postinjection for the toxin-binding inhibition assay. The mice received 2 μg of the Alexa Fluor 555-conjugated typhoid toxoid ± 8 μg of the indicated VHH antibody. The pH2AX signals, reflecting host cell DNA damage repair responses, were measured 6 days after the toxin challenge.

### (ii) Frozen tissue preparation.

The mice were anesthetized with isoflurane, followed by perfusion sacrifice, which was conducted by sequentially administering 50 mL of each 10% sucrose and 4% paraformaldehyde, respectively. The brain samples were extracted, fixed in 4% paraformaldehyde for 24 h at 4°C, washed with PBS, and immersed in 30% sucrose solution overnight for cryoprotection. The brain tissues were trimmed for coronal sections and placed in cassettes for Tissue-Tek optimum cutting temperature (OCT) embedding. The embedded tissue sections were flash frozen in isopentane cooled to −80°C. Tissue samples were cut to be 8 μm thick and stored at −80°C until staining.

### (iii) Immunofluorescence staining.

The frozen tissue sections were fixed with 4% paraformaldehyde for 10 min, washed with PBS-T, and blocked in 3% BSA-PBS for 30 min. The primary antibodies, anti-γ-H2AX (catalog no. PA5-77995; Invitrogen; 1:100) and CD31 (BD Bioscience; clone MEC 13.3; 1:100), were added to the tissue sections and incubated overnight at 4°C. The sections were washed with PBS-T and incubated with the indicated fluorochrome-conjugated secondary antibodies (Molecular Probes) for 1 h at room temperature in the dark. The nuclei were counterstained with DAPI (4′,6- diamidino-2-phenylindole), and the slides were mounted in an antifade mounting solution (Electron Microscopy Sciences). Immunofluorescent images were acquired using a Leica DMI6000B/DFC340 FX fluorescence microscope system. For image acquisition, 1,600- by 1,200-pixel full-frame pictures of various channels were recorded as 16-bit TIFF files with ×40 or ×100 magnification. To quantify the fluorescence signal (Fig. 5), we calculated the corrected total cell fluorescence (CTCF) using ImageJ. The formula for CTCF calculation was CTCF = integrated density − (area of total cell × mean fluorescence of background). For each group, a minimum of 20 images from one experiment were taken, and the results of two independent experiments were plotted as a bar graph.

### Quantification and statistical analysis.

Data were tested for statistical significance with GraphPad Prism software. The number of replicates for each experiment and the statistical test performed are indicated in the figure legends. ImageJ was used to analyze microscopy images and FlowJo for flow cytometry data.

### Data availability.

Data will be made publicly available upon publication and upon request for peer review.
