# Supplementary material for: Neutralization of Typhoid Toxin by Alpaca-Derived, Single-Domain Antibodies Targeting the PltB and CdtB Subunits
Source: Infect Immun. 2022 Feb 17;90(2):e00515-21. doi: 10.1128/iai.00515-21 (PMC8852740; doi:10.1128/iai.00515-21)
Supplement: Supplemental file 1 — Supplemental material. Download iai.00515-21-s0001.pdf, PDF file, 0.4 MB [file iai.00515-21-s0001.pdf]

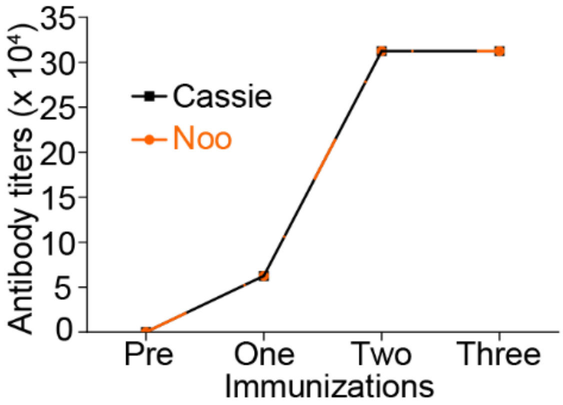

S. Figure 1, related to Fig. 1. ELISA analysis of alpaca sera immunized with typhoid toxoid. Serum reciprocal endpoint titers were >100,000 after two immunizations in both Cassie and Noo alpacas.

```
T2C1            QVQLVETGGGLVQAGGSLRLSCAASGRTFSGDAMGWFRQAPGKEREFVAAISWNGGSTDY
T2G4            QVQLVESGG------GSLRLSCAVFGRTFSGDAVGWFRQAPGKEREFVAAINWNGRSTDY
T2B6            QLQLAESGGGLVQAGDSLRLSCADSGRTFSDYAMGWFRQAPGKEREFVAAISWNGRSTYY
T2A1            QVQLVESGGGLVQAGGSLRLTCAGSGRTFSGDVMGWFRQAPGKEREFVAAISYNGGSTYY
                *:**.*:**      .****:**  *****. .:*****************.:** ** *

T2C1            ADSVKGRFTISR-DAKNTVYLQMNSLKPEDTAVYYCASSREDYGSSFLRGSYDYLGQGTQ
T2G4            ADSVKGRFTISRDDTENTVYLQMNSLKPEDTAVYYCATSREDYGSSFLRGSYDYLGQGTQ
T2B6            IEAVKDRFTISRDNAKKTLYLQMNSLKPEDTAVYYCAAAREDYGSSLVKGSADYWGQGTQ
T2A1            ADSVKGRFTISRDNAKNTVYLQMDGLKPGDTAVYYCASSREDYGSSLAKGSYDYWGQGTQ
                 ::**.****** ::::*:****:.*** ********::*******: :** ** *****

T2C1            VTVSSAHHSEDPS
T2G4            VTVSSAHHSEDPS
T2B6            VTVSSAHHSEDPS
T2A1            VTVSSEPKTPKPQ
                *****  :: .*.
```

S. Figure 2, related to Fig. 1. Amino acid sequence comparison analysis of anti-PltB VHH antibodies in the T2G4 family. The VHH family assignment was completed based on their CDR3 similarity using DNASTAR Lasergene. This figure was prepared using ClustalW.

```
T2A2            QVQLAETGGGLVQPGGSLRLSCAVSGFMVNSYDIGWFRQAPGKEREGVSCISSSGGATHY
T2F6            QLQLAESGGGLVQPGGSLRLSCAVSGFMVNSYDIGWFRQAPGKAREGISCINTSGDTPNY
                *:****:************************************ ***:***.:**.:.:*

T2A2            VDSVKGRFTISRDNAKNMVYLQMNSLKPEDTATYYCAANRMTRCSDYHGYPFWGQGTQVT
T2F6            ADSVKGRFTISRDNAKNMVYLQMNSLKPEDTATYFCAANRMTRCSDYNGYPYWGQGTQVI
                .*********************************:************:***:*******

T2A2            VSSTHHSEDPS
T2F6            VSSAHHSEDPS
                ***:*******
```

S. Figure 3, related to Fig. 1. Amino acid sequence comparison analysis of anti-PltB VHH antibodies in the T2F6 family. The VHH family assignment was completed based on their CDR3 similarity using DNASTAR Lasergene. This figure was prepared using ClustalW.

```
T2E8            QVQLAESGGGSVQAGGSLRLSCAASGLTFDKYAIGWFRQAPGKGREMVSCINTNYGSTYY
T2G1            QVQLAESGGGLVQAGGSLRLSCAASGLTFDKYAIGWFRQAPGKERELVSCISTNYGSTYY
T2A3            QVQLAESGGGLVQAGGSLRLSCAASGLTFDKYAIGWFRQAPGKERELVSCISTNYGSPYY
T2A7            QVQLVETGGGLVQTGGSLRLSCAASGFTFDDFAIGWFRQAPGKEREWVSCISSRNGSPYY
                ****.*:*** **:************:***.:*********** ** ****.:. **.**

T2E8            ADSVKGRFTISSDTAKNTVYLQMNSLSPEDTAVYYCAADTRSSWPSCNALDTWGQGTLVT
T2G1            ADSVKGRFTISTDNAKNTVYLQMNGLKPEDTAVYYCAADTRSSWPSCNALDTWGQGTLVT
T2A3            ADSVKGRFTISSDNAKNTVYLQMNSLKPEDTAVYYCAADTRSSWPSCNALDARGQGTLVT
T2A7            ADSVKGRFTISSDNAKNTVYLQMNSLKPEDTAVYYCKADTDSIVPSCNALDTWGQGTLVT
                ***********:*.**********.*.********* *** *  *******: *******

T2E8            VSSAHHSEDPS
T2G1            VSSAHHSEDPS
T2A3            VSSEPKTPKPQ
T2A7            VSSAHHSEDPS
                ***  :: .*.
```

S. Figure 4, related to Fig. 1. Amino acid sequence comparison analysis of anti-PltB VHH antibodies in the T2A7 family. The VHH family assignment was completed based on their CDR3 similarity using DNASTAR Lasergene. This figure was prepared using ClustalW.

```
T2B2            QLQLAESGGGLVQAGGSLRLSCAASGRTFNTLHLAWFRQAPGNERDFVASISGSLGSLHY
T2G11           QVQLVETGG-LVQAGGSLSLSCAVSGRTFNLLHLAWFRQAPGNERDLVASISGSLGSVYY
                *:**.*:** ******** ****.****** ***************:**********::*

T2B2            ADSVKGRFTISRDNANNTVYLQMNSLKLEDTAVYYCAGAPIYDRFSSSSSSYDYWGQGTQ
T2G11           ADSVRGRFTISRDAANNTVYLQMNSLKLEDTAVYYCAGAPIFDRFSSSSSSYDYWGQGTQ
                ****:******** ***************************:******************

T2B2            VTVSSAHHSEDPS
T2G11           VTVSSAHHSEDPS
                *************
```

S. Figure 5, related to Fig. 1. Amino acid sequence comparison analysis of anti-PltB VHH antibodies in the T2G11 family. The VHH family assignment was completed based on their CDR3 similarity using DNASTAR Lasergene. This figure was prepared using ClustalW.

```
T2B4            QVQLVETGGALVQTGGSLRLSCAVSGRSSSTQGLGWYRQAPGKEREFVATINWISGATYY
T2F3            QVQLAETGGALVQTGGSLRLSCAVSGRNSEIQGLGWYRQAPGKEREFVATINWRSGATYY
                ****.**********************.*. ********************** ******

T2B4            SDSVKGRFSISRDNAENTVSLQMHDLKPDDTAVYYCGAGLSPIARMDYWGQGTQVTVSSE
T2F3            DDSVKGRFSISRDNAENTVSLQMNDLKPDDTAVYYCGAGMSPIARMDYWGQGTQVTVSSA
                .**********************:***************:******************* 

T2B4            PKTPKPQ
T2F3            HHSEDPS
                 :: .*.
```

S. Figure 6, related to Fig. 1. Amino acid sequence comparison analysis of anti-PltB VHH antibodies in the T2B4 family. The VHH family assignment was completed based on their CDR3 similarity using DNASTAR Lasergene. This figure was prepared using ClustalW.

```
T2D2            QVQLAETGGGLVQPGGSLRLSCAASGFDFSRSTMSWVRQAPGKNIEWVSDINSGGGLTNY
T2F7            QVQLVESGGGLVQPGGSLRLSCAASGFDFSRSTLSWVRQSPGKDFEWVSDINSGSTLTNY
                ****.*:**************************:*****:***::*********. ****

T2D2            GDTVKDRFTISRDNAKNTLYLQMNSLDVEDTAVYYCVKGMWITSDQPRGQGTQVTVSSAH
T2F7            GDSVKGRFTTSRDNAKNTLYLQMNSLKPEDTAVYYCVKGMWITRDQPRGQGTQVTVSSAH
                **:**.*** ****************. *************** ****************

T2D2            HSEDPS
T2F7            HSEDPS
                ******
```

S. Figure 7, related to Fig. 1. Amino acid sequence comparison analysis of anti-PltB VHH antibodies in the T2D2 family. The VHH family assignment was completed based on their CDR3 similarity using DNASTAR Lasergene. This figure was prepared using ClustalW.

```
T2D9            QLQLVESGGGLVQPGGSLRLSCVLSGSDLAYYNVGWFRQAPGKEREGVACTSRVSDTKYY
T2F11           QVQLVETGGGLVQPGGSLRLSCVLSGSDLAYYNVGWFRQAPGKEREGVACTSRVSDTKYY
                *:****:*****************************************************

T2D9            ADSVKARFTVARDNAKNTVNLQMSSLRPEDTAVYSCALLRWCTTEDSDSEGMGHWGKGTL
T2F11           ADSVKARFTVARDNAKNTVNLQMNSLRPEDTAVYSCALLRWCTTEGSDSEGMGHWGKGTL
                ***********************.*********************.**************

T2D9            VTVSSEPKTPKPQ
T2F11           VTVSSEPKTPKPQ
                *************
```

S. Figure 8, related to Fig. 1. Amino acid sequence comparison analysis of anti-PltB VHH antibodies in the T2D9 family. The VHH family assignment was completed based on their CDR3 similarity using DNASTAR Lasergene. This figure was prepared using ClustalW.

```
T2D10           QVQLAETGGGLVQAGGSLRLSCAASGLTSSRYAMAWFRQVPGLEREFVASMTGSGGRTFY
T2F2            QVQLAESGGGLVQAGGSLRLSCVASGRTFSSYAMGWFRRAPGKEREFVASILWSDGSAAY
                ******:***************.*** * * ***.***:.** *******:  *.* : *

T2D10           ADSVKGRFTISRDNAKNTVYLQMNNLNPEDTAVYYCAVEYRDSGTRWSNEYDYWGQGTQV
T2F2            ADSVEGRFTISRDNAKNTVHLQMNSLQPEDTAVYYCAAEARGSGTRWGNEYDYWGQGTQV
                ****:**************:****.*:**********.* *.*****.************

T2D10           TVSSEPKTPKPQ
T2F2            TVSSAHHSEDPS
                ****  :: .*.
```

S. Figure 9, related to Fig. 1. Amino acid sequence comparison analysis of anti-PltB VHH antibodies in the T2D10 family. The VHH family assignment was completed based on their CDR3 similarity using DNASTAR Lasergene. This figure was prepared using ClustalW.

```
T2G6            QVQLVESGGGLVQAGGSLRLSCAASGFTFDDYAIGWFRQAPGKEREMILCINSRSGSTSY
T2H1            QVQLVESGGGLVQAGGSLRLSCAASGFTFDDYAIGWFRQAPGKEREQILCINSNRARTSY
T2G7            QVQLVESGGGSVQAGGSLRLSCAASGFTFDDYAIGWFRQAPGKEREMILCINSRAGMTSY
T2G9            QVQLVETGGGSVQAGGSLRLSCAASGFTFDDYAIGWFRQAPGKEREMILCINSRGGSTSY
T2H5            QVQLVETGGGLVQAGGSLRLSCTASGFTFDEYAIGWFRQAPGKEREMILCISSVGG-STY
T2E12           QVQLAESGGGLVQAGGSLRLSCAASGFTFDDYAVAWFRQAPGKAREMVLCISSSGG-SSY
                ****.*:*** ***********:*******:**:.******** ** :***.*  . ::*

T2G6            YADSVKGRFTVSVNSAKNTAYLQMNSLKPEDTAVYYCAADVTHPHSCNALDAWGQGTLVT
T2H1            YAESIKGRFTVSVNSAKNTVYLQMNSLKPEDTAVYYCAADVTHPHSCNALDAWGQGTLVT
T2G7            SADSVKGRFTVSVNSAKNTAYLQMNSLKPEDTAKYYCAADVTHPHSCNALDAWGQGTLVT
T2G9            YADSVKGRFTVSVNSAKNTAYLQMNSLKPEDTAVYYCAADVTPPHSCNALDAWGQGTLVT
T2H5            YADSVKGRFTISSDNAKNTMYLQMSSLKPEDTAKYYCAVDVTPPHSCNALDTWGQGTLVT
T2E12           YADFVKGRFTVPSNSAKNTVYLQMNSLKPEDTAKYICAVDATYPHSCNALDAWGQGTLVT
                 *: :*****:. :.**** ****.******** * **.*.* ********:********

T2G6            VSSAHHSEDPS
T2H1            VSSAHHSEDPS
T2G7            VSSAHHSEDPS
T2G9            VSSEPKTPKPQ
T2H5            VSSAHHSEDPS
T2E12           VSSEPKTPKPQ
                ***  :: .*.
```

S. Figure 10, related to Fig. 1. Amino acid sequence comparison analysis of anti-PltB VHH antibodies in the T2E12 family. The VHH family assignment was completed based on their CDR3 similarity using DNASTAR Lasergene. This figure was prepared using ClustalW.

```
T2E1        QVQLAESGGGFVQAGGSLRLSCVESGRSFSVFAMGWFRQAPGKERELVAAIS-WRGDSTY
T2C7        QLQLAESGGGLVQAGGSLRLSCVVSGRTDSSYAVGWFRQAPGKEREFVAAIM-WSGISTY
T2B12       QLQLVESGGGSVQAGGSLRLSCAASGFTFGDYAIAFFRQAPGKEREGVSCIS-TYDGSTY
T2E7        QLQLVESGGGLVQPGGSLRLSCAASGFTLDYYAIGWFRQAPGKERERVSCIS-GRDGGTY
T2D8        QLQLVESGGGLVQPGGSLRLSCAASGFTLDYYAIGWFRQAPGKEREWVSCLGRSSSSTSY
T2E11       QLQLVETGGGLVQPGGSLRLSCAASGFSLDYYAIGWFRQAPGKEREGVGCIS-SSGSTTK
T2C5        QVQLVETGGGLVQPGGSLRLSCATSGFTFSSTTMTWVRQAPGKGLEWLSGIY--SDGRTE
T2H3        QVQLAESGGGLVQAGGSLRLSCVASGRTFDINTMAWYRQAPGKQRDFVASSY--HGGITN
            *:**.*:*** **.********. ** : .  :: : ******  : :.      .  :

T2E1        YADLVKGRFTISRDNAKNAVYLQMNSLKPEDTAVYYCAADRSDYVVTHEYDY----WGQG
T2C7        YADSVKGRFTISLDNAKSTVYVQMNSLKPEDTAVYYCAAWKG--ATVTDDDP----WGQG
T2B12       YGDSVKGRFTISSDNAKNTVYLQMNSLKPEDTAVYYCAADRAD--CGWYYPY--DYWGQG
T2E7        YADSVKGRFAISRDNAQNTVYLQMSRLKPEDTAAYYCAADYADNYCTGSFPP--DYWGQG
T2D8        VADSVQGRFTISRDNAKNTVYLQMNSLKPEDTAVYYCAADLSS-QISSCNAL--DTWGQG
T2E11       YADSVKGRFTISRDNAKNTVYLQMNSLKPEDTAVYYCAARNPADLNCGAYVLNGEYWGQG
T2C5        YADSVKGRFTISRDNAKNTLYLEMNSLKPEDTAVYFCAKGLLG-----------AERGQG
T2H3        YADSVKGRFTMSRDNAKNTVYLQMNSLKPEDTGVYYCTWRGGG-----------VYWGQG
             .* *:***::* ***:.::*::*. ******..*:*:                   ***

T2E1        TQVTISPEPKTPKPQ
T2C7        TQVTVSSEPKTPKPQ
T2B12       TQVTVSSEPKTPKPQ
T2E7        TQVTVSSAHHSEDPS
T2D8        TLVTVSSEPKTPKPQ
T2E11       TQVTVPSEPKTPKPQ
T2C5        TRVTVSSEPKTPKPQ
T2H3        TQVTVSSEPKTPKPQ
            * **:..  :: .*.
```

S. Figure 11, related to Fig. 1. Amino acid sequence comparison analysis of anti-PltB VHH antibodies in the T2E1 and unique families. The VHH family assignment was completed based on their CDR3 similarity using DNASTAR Lasergene. This figure was prepared using ClustalW. Note that only T2E1 in the family was characterized in the study.

```
T4B5      QLQLAESGGGLVQPGGSLRLSCAASGSIFSINVMGWYRQVPGKQREWVASITRGGGTLYADSVKGRFTIS
T4C4      QVQLAESGGGLVQPGGSLRLSCAASGSIFSINVMGWYRQAPGKQRESVAVITRGGGTMYADSVKGRATIS
T4C12     QVQLAESGGGLVQPGGSLRLSCAASGSIFSINVMGWYRQAPGKQREFVAVITRGGSTDYADSVKGRFTIS
T4C11     QLQLVETGGGLVQPGGSLRLSCAASGSIFSINIMGWYRQAPGKQRESVAVITRGGGTMYADSVKGRFTIS
T4E5      QLQLVETGGGLVQPGGSLKLSCVASGSIFSINVMGWFRQASGKQREVVAQITRGGGTIYADSVKGRFTIS
T4E8      QLQLVESGGGLVQAGGSLRLSCAASGMSFSTYTMGWYRQAPGKRRGLVATISSGGSTNYADSVKGRFTIS
T4F3      QLQLVETGGGLVQPGGSLTLSCAASGFTLNYWHMGWYRQAPGKQRELVATISGHGITNYADSVKGRFTIS
          *:**.*:******.**** ***.***  :.   ***:**..**:*  ** *:  * * ******** ***

T4B5      RDSAKNTVYLQMNSLKPEDTAVYYCNADLMEDSDSDYHEYWGQGTQVTVSSAHHSEDPS
T4C4      RDSAKNTVYLQMNSLKPEDTAVYYCNADLMQAVDSEYEDYWGQGTQVTVSSAHHSEDPS
T4C12     RDNAKNTVYLQMNSLKPEDTAVYYCNADLMQTSASEYEDCWGQGTQVTVSSEPKTPKPQ
T4C11     RDSAKNTVYLQMNSLKPEDTAVYYCNADLMQAVDSEYEDYWGQGTQVTVSSEPKTPKPQ
T4E5      RDDAKNSVYLQMNSLNPEDTAVYLCNADLMQGIDSEYEDYWGQGTQVTVSSEPKTPKPQ
T4E8      RDNAKDTVYLQMNSLKPEDTAEYYCNVKGDYQTPN--MGYWGQGTRVTVSSEPKTPKPQ
T4F3      RDDAKNSVYLQMNSLKPEDTAVYYCNFISRTN-------DWGQGTQVTVSSEPKTPKPQ
          **.**::********:***** * **              *****:*****  :: .*.
```

S. Figure 12, related to Fig. 1. Amino acid sequence comparison analysis of anti-CdtB VHH antibodies in the T4B5 and unique families. The VHH family assignment was completed based on their CDR3 similarity using DNASTAR Lasergene. This figure was prepared using ClustalW.

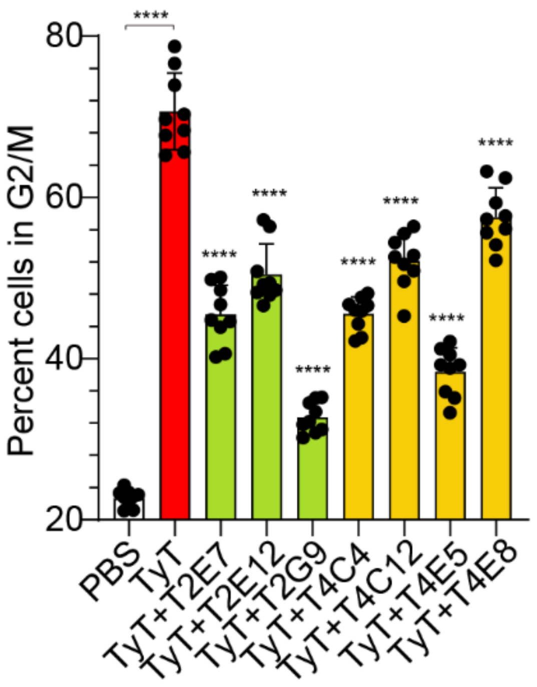

S. Figure 13, related to Fig. 1. Typhoid toxin neutralization by VHH antibodies. Percent cells in G2/M are shown to indicate typhoid toxin-induced cellular toxicities. Henle-407 cells were treated with PBS, typhoid toxin (TyT, 1.2 pM), or a mixture of TyT and each VHH indicated (1:400) for 66 hrs. Cell cycle profiles were analyzed via flow cytometry. Three independent experiments were performed. Bars represent average ± SD. ****, $p<0.0001$ compared to TyT. N=9 per group. Unpaired two-tailed t-tests.

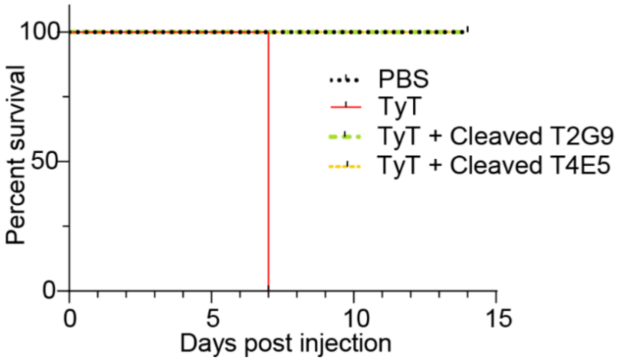

S. Figure 14, related to Fig. 4. Percent survival of mice received typhoid toxin with or without VHH. Groups of Cmah null mice were administered with typhoid toxin (2 µg) with or without VHH (8 µg). This data confirms that the *in vivo* toxin neutralization effects of VHHs are indeed due to the VHHs. Cleaved T2G9 and cleaved T4E5 contain VHH only without an epitope tag. N=2.

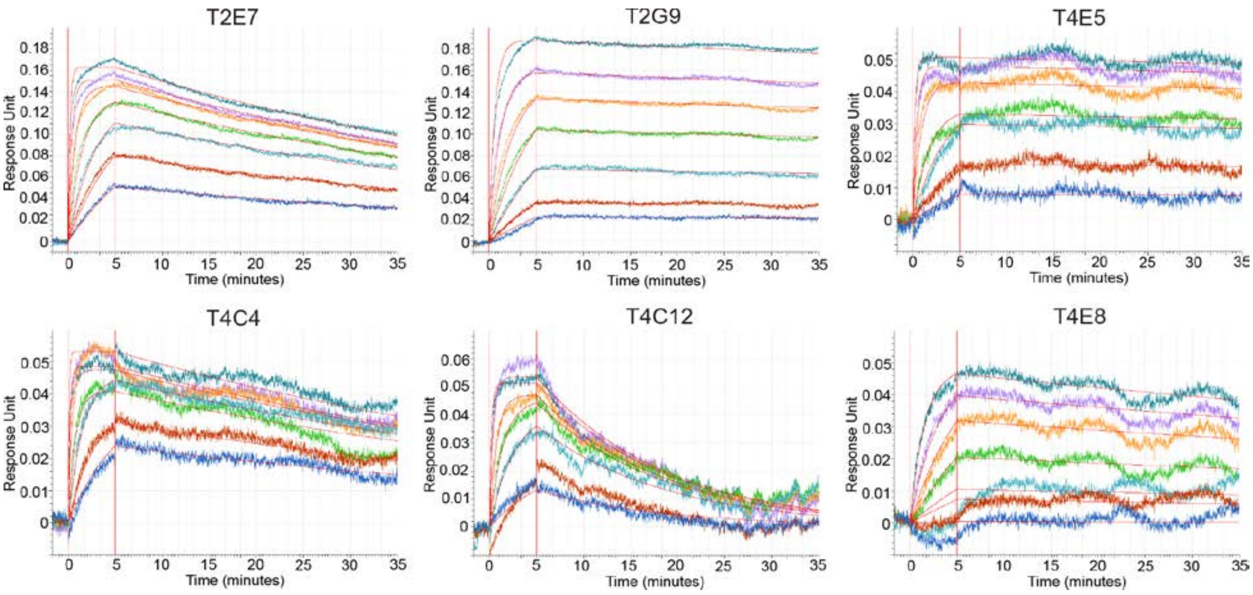

S. Figure 15, related to Table 1. Octet affinity sensorgrams. The affinity of each VHH was measured on an Octet RED96e Biolayer Interferometer at 25ºC. Biotinylated Typhoid Toxin was immobilized on Streptavidin-coated sensors, and VHH kinetics were tested at concentrations (in nM) of 200 (teal), 100 (purple), 50 (orange), 25 (green), 12.5 (cyan), 6.25 (red) and 3.125 (blue). An eighth typhoid toxin-coated sensor was dipped into the buffer with no VHH and its signal was subtracted from all VHH samples to account for background drift. A 1:1 model of VHH:Toxin interaction was used to model the kinetics of each interaction (red lines).

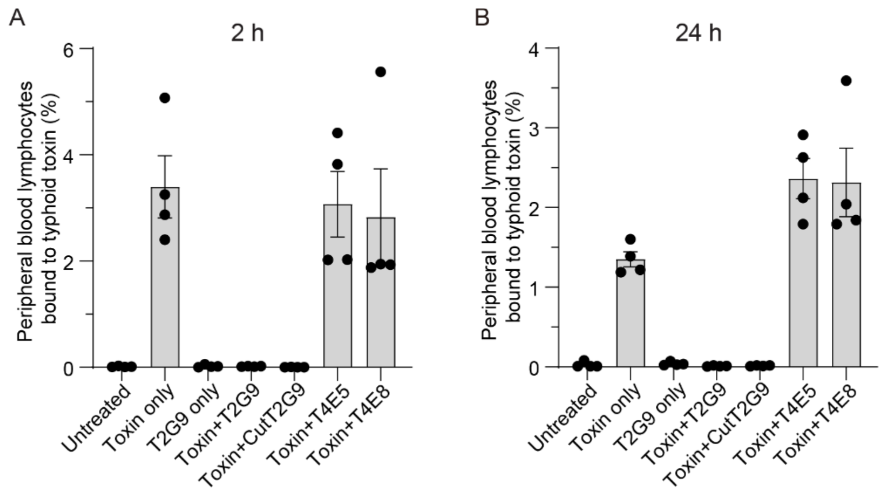

S. Figure 16, related to Fig. 4. Percentage of peripheral blood lymphocytes bound to typhoid toxin at 2 or 24 hrs. Groups of Cmah null mice (n=4) were administered with typhoid toxin conjugated to Alexa Fluor 555 (2 µg) with or without VHH (8 µg). Blood was drawn 2 or 24 hrs after administration of toxin, VHH, or toxin complexed with VHH. N=4. Two independent experiments.
